# Cardiac Remodeling and Diastolic Dysfunction in Paroxysmal Atrial Fibrillation

**DOI:** 10.3390/jcm10173894

**Published:** 2021-08-30

**Authors:** Nathalie Noirclerc, Olivier Huttin, Christian de Chillou, Christine Selton-Suty, Laura Fillipetti, Jean Marc Sellal, Erwan Bozec, Erwan Donal, Zohra Lamiral, Masatake Kobayashi, João Pedro Ferreira, Patrick Rossignol, Nicolas Girerd

**Affiliations:** 1 Department of Cardiology, University Hospital of Nancy, 54000 Nancy, France; n.noirclerc@chru-nancy.fr (N.N.); c.dechillou@chru-nancy.fr (C.d.C.); c.seltonsuty@chru-nancy.fr (C.S.-S.); l.Fillipetti@chru-nancy.fr (L.F.); jm.sellal@chru-nancy.fr (J.M.S.); 2 Université de Lorraine, Inserm, Centre d’Investigations Cliniques-1433 and Inserm U1116, CHRU Nancy, F-CRIN INI-CRCT, 54000 Nancy, France; e.bozec@chru-nancy.fr (E.B.); z.lamiral@chru-nancy.fr (Z.L.); mkoba3858@gmail.com (M.K.); jp.ferreira@chru-nancy.fr (J.P.F.); p.rossignol@chru-nancy.fr (P.R.); n.girerd@chru-nancy.fr (N.G.); 3 Département de Cardiologie & CIC-IT U 804, Centre Hospitalier Universitaire de Rennes, 35033 Rennes, France; e.donal@chu-rennes.fr

**Keywords:** atrial fibrillation, diastolic dysfunction, left atrial remodeling, echocardiography, heart failure

## Abstract

Background: Atrial fibrillation (AF) leads to the development of cardiac remodeling/diastolic dysfunction and vice versa. We intended to determine whether cardiac remodeling/diastolic dysfunction is present at early stages of AF. Methods: We studied 175 patients with paroxysmal AF, compared with 175 matched control subjects, who had available echocardiography data to investigate the association between echocardiographic variables and AF from the STANISLAS cohort. Results: In this study (mean age 55 years; 70.3% male), patients with paroxysmal AF had greater left ventricular mass compared to matched controls (*p* < 0.05). Patients with paroxysmal AF were also likely to have larger left atrial volume and a higher peak tricuspid regurgitation velocity, leading to higher prevalence (though <10% in the AF group) of diastolic dysfunction (all-*p* < 0.05). Multivariable conditional logistic regression models showed that paroxysmal AF was significantly associated with increased left ventricular mass and left atrial enlargement (all-*p* < 0.001), but not with e’ and deceleration time of E wave (all-*p* > 0.1). Conclusions: Left ventricular mass and left atrial enlargement rather than diastolic dysfunction (as evaluated by echocardiography) were associated with paroxysmal AF irrespective of body mass index, blood pressure and renal function. These findings suggest that cardiac remodeling may occur very early in the natural history of AF.

## 1. Introduction

Atrial fibrillation (AF) is the most common arrhythmia. It is associated with incident heart failure (HF), and with its progression [1]. It has been shown to have important risk-stratification properties in the setting of primary prevention and interacts with other important CV variables such as valvular calcifications [2].

AF causes the progressive development of left atrial (LA) remodeling, particularly when AF is persistent. Patients with pre-existing structural cardiac abnormalities such as LA enlargement are likely to have a high incidence of AF, especially in subjects with cardiovascular risk factors or in patients with HF. Thus, AF and atrial enlargement are inter-related in a deleterious loop. Their common background and associated risk factors (i.e., aging, obesity, diabetes, and hypertension) suggest that they share etiologies [3]. However, which abnormalities, AF or LA enlargement, proceed the other is yet to be studied. Precise cardiac phenotyping in paroxysmal AF might identify structural or functional abnormalities that precede the development of persistent AF.

## 2. Methods

We used echocardiographic data to investigate cardiac structure and diastolic function in 175 consecutive patients with paroxysmal AF, compared with 175 matched control subjects from the STANISLAS Cohort [4,5,6]. Patients with paroxysmal AF had no known or overt HF. They were enrolled in the current study after hospitalization to our center for a first AF ablation, when they were in sinus rhythm; their AF burden was not known. Controls were matched for age, sex and a history of hypertension. All subjects underwent echocardiography according to the current EAE/ASE recommendations, and diastolic dysfunction (DD) was diagnosed if ≥2 of 4 echocardiographic markers were abnormal [e’ velocity, E/e’, LA volume index, and peak tricuspid regurgitation (TR) velocity] [4]. We also assessed the utility in this predominantly middle-aged population of the recently proposed LA volume index threshold of 25 mL/m^2^ [7]. Statistical comparisons were performed using non-parametric tests. Associations of echocardiographic variables with paroxysmal AF were analyzed using conditional logistic regression models, taking account of the matched nature of the data, after adjustment for potential covariates including estimated glomerular filtration rate (eGFR) and body mass index (BMI).

## 3. Results

Patients with paroxysmal AF had higher BMI, poorer renal function, more severe dyspnea, a higher left ventricular (LV) mass, and a greater proportion of LA remodeling, compared with matched controls (Table 1). Patients with paroxysmal AF were also more likely to have DD (9.7% vs. 2.3%, *p* = 0.0003), although most Doppler diastolic parameters (lateral e’, E/e’ and deceleration time) were not significantly different compared with controls. The difference in DD was associated with higher prevalence of LA volume index > 34 mL/m² and TR > 2.7 m/s in the patients with paroxysmal AF, at 40.8% and 29.3%, respectively, compared with <10% in the controls (both, *p*-value < 0.05).

In multivariable analysis accounting for the matching and adjusted for BMI and eGFR, LV mass index (LVMi) [OR(95%CI) = 4.52 (2.05–9.97), *p* < 0.0002) and LA remodeling defined as LA volume index (LAVI) > 25 mL/m^2^ [OR = 5.04 (2.55–9.99), *p* < 0.0001] were significantly associated with paroxysmal AF whereas e’ and DT were not (Table 2). The current definition of DD was significantly associated with AF (Table 2) though it was present in less than 10% of patients with AF (Table 1).

## 4. Discussion

In this study, only 10% of patients with paroxysmal AF had DD as defined by the recent recommendations. The independent differences in echocardiographic variables between patients with paroxysmal AF and controls were related mainly to LV/LA remodeling and increased TR, rather than to e’ velocities or E/e’. Since the groups had similar hypertension status and systolic blood pressures, we could disregard any influence of increased afterload. The much higher prevalence of atrial and ventricular remodeling, rather than DD, suggests that LV hypertrophy and LA enlargement may precede persisting AF rather than being the ultimate consequence of persisting AF. This finding may be the consequence of atrial myopathy being both the substrate and the outcome of atrial fibrillation [8]; following this line of reasoning, stress-induced atrial myopathy would be the primum movens of structural and electrical atrial remodeling, resulting in AF. From a pathophysiological standpoint, growing evidence exists regarding the links between atrial fibrosis and AF. Wang et al. recently showed that a mutation targeting MFAP4 attenuates LA remodeling and susceptibility to AF [9]; This result suggests that common upstream pathways are involved in both atrial fibrosis/remodeling and AF.

Yet, we did also identify LV hypertrophy as being more frequent in paroxysmal AF, which suggests that pathophysiological processes going beyond the atria are involved in a number of patients. This finding is in line with the report of Seko et al., reporting an increased prevalence of AF appears with increasingly abnormal LV remodeling patterns [10].

Our study could also support the concept that paroxysmal AF precedes the development of DD in most patients with paroxysmal AF, at least when DD is defined using the cut-points for e’ velocities and E/e’ proposed in the 2016 recommendations. Indeed, a minority of patients with AF had DD (<10%), which suggests that DD (as defined by the current echocardiographic definition) is hardly present in this population. The e’ velocity was abnormal in only 23.1% of the patients with paroxysmal AF, and none had an abnormal E/e’ index.

Although the prevalence of DD, LA remodeling and LV remodeling were different in our cohort, the associations of all three parameters with AF in the multivariable model were similar (OR ~5). Another intriguing point is the greater difference in TR velocity than in mitral flow or myocardial velocities between paroxysmal AF and controls, suggesting that an increased TR velocity might be clinically useful, albeit infrequent, and may constitute an early sign of disease rather than just a late consequence of chronically elevated LV filling pressures. A growing body of evidence suggests that, not infrequently, isolated functional TR is concomitant to AF and right atrial remodeling [11]. This further supports the early involvement of TR in the natural history of AF.

## 5. Strengths and Limitations

The main strengths of our study are the use of research-quality echocardiography in a community-based cohort (STANISLAS), and the refined methods (matching and adjustment) used to evaluate the association of echocardiographic variables with paroxysmal AF. However, it is a single-center study of patients referred for ablation of paroxysmal AF, who are not representative of the much broader population of all patients with AF. In addition, TR and DD could not be included in the multivariable analysis because the number of subjects with these parameters was small. We applied the most recent definition of DD, but have emphasized how changing definitions strongly influences the proportion of patients who are diagnosed as abnormal [4]. Other definitions of DD may have yield different results and further analysis should be conducted with future definition of DD. In addition, as DD goes beyond its echocardiographic definition, our results do not formally ascertain that DD is not involved in early stages of AF.

## 6. Conclusions

In the current study, LA enlargement and LV hypertrophy, rather than LV diastolic dysfunction, are associated with paroxysmal AF, irrespective of hypertension history, blood pressure, eGFR and BMI. We found also that very few patients with AF fulfill the current criteria for DD. These results suggest that cardiac remodeling occurs very early in the natural history of AF, before it progresses from paroxysmal to persistent AF and before the development of DD.

## Figures and Tables

**Table 1:** Clinical and echocardiographic characteristics of controls and patients with paroxysmal atrial fibrillation.

	Control Subjects *n* = 175	Paroxysmal AF Subjects *n* = 175	*p*-Value
CLINICAL CHARACTERISTICS	Mean ± SD	Mean ± SD	
Age (years)	55 ± 11	55 ± 11	matched
Male gender (%)	123 (70.3%)	123 (70.3%)	matched
Arterial hypertension self-reported by patient	68 (38.9%)	68 (38.9%)	matched
Systolic blood pressure (mmHg)	130 ± 16	130 ± 21	0.47
BMI (Kg/m^2^)	26 ± 4	27 ± 5	0.049 *
Diabetes Mellitus	15 (8.6%)	15 (8.6%)	1.00
Heart rate (bpm)	63 ± 8	64 ± 13	0.12
eGFR (MDRD mL/min/1.73 m2)	94 ± 15	78 ± 21	<0.0001 *
Dyspnea	29 (16.7%)	47 (29.9%)	0.005
**ECHO CHARACTERISTICS**			
LV mass index ASE Mean (g/m^2^)	77.8 ± 18.2	93.5 ± 29.9	<0.0001
>88 in women and > 102 g/m2 in men	17 (10.8%)	62 (40.8%)	<0.0001
LVEF (%)	65.9 ± 5.9	62.7 ± 7.2	<0.0001
LV End-diastolic volume (mL/m²)	48.3 ± 12.5	47.3 ± 12.2	0.60
Left atrial volume index Mean (mL/m²)	22.3 ± 6.8	30.45 ± 11.94	<0.0001
>34 mL/m²	11 (6.3%)	49 (29.3%)	<0.0001
>25 mL/m^2^	50 (28.7%)	93 (62.4%)	<0.0001
E/A ratio	1.1 ± 0.3	1.23 ± 0.50	0.010
Deceleration time (DT) Mean (ms)	210.0 ± 51.5	201.5 ± 65.7	0.063
<160 (ms)	27 (15.4%)	40 (23.4%)	0.10
e’ lateral Mean (cm/s)	11.5 ± 3.3	11.7 ± 3.6	0.68
<10 cm/s	51 (29.3%)	33 (23.1%)	0.46
Estimated pulmonary arterial pressure (mmHg)	18.8 ± 5.3	24.8 ± 6.5	<0.0001
TR > 2.7 m/sec	2 (2.7%)	14 (17.5%)	0.014
Average E/e’ Mean	9.01 ± 2.42	9.15 ± 2.61	0.42
>14	2 (1.2%)	0	----
**DIASTOLIC FUNCTION CLASSIFICATION**			
Normal diastolic function <50%	166 (94.9%)	138 (78.9%)	
Indeterminate (50% positive)	5 (2.9%)	20 (11.4%)	0.0003
Diastolic Dysfunction (>50%)	4 (2.2%)	17 (9.7%)	

* Further adjustment for conditional logistic regression models.

**Table 2:** Association of diastolic dysfunction, diastolic functional parameters (e’ or deceleration time) and cardiac remodeling variables (LVMI/LAVI) with paroxysmal atrial fibrillation status in multivariable conditional logistic regression models.

	OR (95% CI)	*p*-Value	P Interaction with Hypertension Status *
DD (Yes vs. No or indeterminate)	5.08 (1.34–19.17)	0.017	0.62
e’ lateral < 10 cm/s	0.71 (0.35–1.41)	0.33	0.55
Deceleration time of E wave < 160 ms	1.18 (0.65–2.15)	0.58	0.73
LVMI > 88 (w) and > 102 g/m2 (m)	4.52 (2.05–9.97)	0.0002	0.59
LAVI > 25 mL/m²	5.04 (2.55–9.99)	<0.0001	0.02 **

Conditional logistic regression models were performed using the matching pairs as strata and adjusted for eGFR < 90 mL/min/1.73 m^2^ and BMI used as a linear variable. * Interaction with hypertension could necessarily not be evaluated in conditional logistic regression as matching was partly based on hypertension status. Interaction models were consequently logistic models (i.e., not conditional models) adjusted for age, sex, hypertension status, eGFR and BMI. ** Association for LAVI > 25 mL/m² in patients with hypertension: 12.51 (4.82–32.44), *p* < 0.0001 and without hypertension: 3.05 (1.57–5.91), *p* = 0.001. Models using TR did not converge and were consequently not provided.

## Data Availability

The data can be made available upon reasonable request to the principal investigator of the study (P.R.).
